# The Treatment of Influenza

**Published:** 1907-07-06

**Authors:** Thomas Wilson Banks

**Affiliations:** Mervyn, Lanark


### THE TREATMENT OF INFLUENZA.

By THOMAS WILSON BANKS, M.B., C.M., Mervyn, Lanark.

Every practitioner has his own way of treating influenza.

I have tried several drugs, such as (1) ammoniated quinine (as recommended by Sir Wm. Broadbent); (2) Dover's powder (the late Sir Grainger Stewart); (3) quin. sulph. (2 gr. every four hours), but I find I get far quicker and better results from—

Sod. salicyl. ... ... ... ... ... gr. xv.
Antifeb. ... ... ... ... ... ... gr. ij.
Ft. pulv. Tales ... ... ... ... iij.
Sig.: Take one every four hours.

This produces profuse perspiration in nearly every case; although I have seen one or two cases unaffected in that way, yet the pains and temperature were relieved to a considerable extent. Three more powders generally produce the desired effect. Most of my patients (and I have treated hundreds) say that it acts like a charm. Headaches, pains all over the body, sore throat, and temperature are all gone in a short time, and although the patients are weak for a day or two they soon gain their usual strength.

I keep them on hot drinks, and four hours after the third powder they require a thorough dry change of clothing followed by a dose of opening medicine. I keep them in bed two days after they feel quite well. Then they begin to get up a little longer each day, and by the end of seven days they are ready for their work.

Dr. Leach, in The Hospital, May 25, 1907, page 208, says that epistaxis may be produced by salicylate of soda, and quotes one case. I have never seen this happen, nor have I ever heard or read of this before. It would be interesting to hear if another such case has happened in the practice of other medical men.

In all the cases that I have treated with salicylate of soda and antifebrin I have not seen any bad effects except a little "singing in the ears" which soon passed off, and I cannot remember having a case followed by complications.

